# Impact of the Coronavirus Disease 2019 Pandemic on Leisure Screen Time and Eating Habits of Japanese High School Students: A Comparison between before and during the Pandemic

**DOI:** 10.3390/healthcare11091265

**Published:** 2023-04-28

**Authors:** Hiromi Inaba, Fumi Hoshino, Kousuke Takano, Misaki Kaiwa, Ayano Kondou, Haruki Ishikawa, Lingzhi Liu, Kazuo Ishigami

**Affiliations:** 1 Center for Nutrition Sciences, Niigata University of Health and Welfare, 1398 Shimami-cho, Kita-ku, Niigata 950-3198, Japan; 2 Department of Health and Nutrition, Faculty of Health Science, Niigata University of Health and Welfare, 1398 Shimami-cho, Kita-ku, Niigata 950-3198, Japan; fumi-h@nuhw.ac.jp; 3 Department of Health Information, Niigata University of Health and Welfare, 1398 Shimami-cho, Kita-ku, Niigata 950-3198, Japan; kousuke-takano@nuhw.ac.jp (K.T.); ishigami@nuhw.ac.jp (K.I.); 4 Majors in Health and Welfare, Graduate School of Health and Welfare, Niigata University of Health and Welfare, 1398 Shimami-cho, Kita-ku, Niigata 950-3198, Japan; hwd20003@nuhw.ac.jp (M.K.); whm22001@nuhw.ac.jp (A.K.); whm22002@nuhw.ac.jp (H.I.); whm22003@nuhw.ac.jp (L.L.)

**Keywords:** COVID-19, adolescents, screen time, eating habit, health

## Abstract

This study aimed to determine whether adolescents’ leisure screen time differed during the coronavirus disease 2019 (COVID-19) pandemic compared to before the pandemic, and to identify factors that affect leisure screen time among Japanese high school students. The Health Behavior in School Children questionnaire was used to investigate differences in eating habits and physical and mental health. The results showed that the leisure screen time of Japanese high school students was 2.6 h (SD = 1.4) before the pandemic, and 3.2 h (SD = 1.5) during the pandemic. The factors that increased leisure screen time were found to differ between boys and girls. No significant deterioration in physical and mental health was observed. The impact of the pandemic on eating habits differed in boys and girls. Boys reported “not feeling great about life” as a factor that increased leisure screen time during the pandemic, suggesting that negative emotions influenced the increase in leisure screen time. The pandemic had a significant impact on girls’ leisure screen time. Longer screen time should be carefully monitored because it can lead to sleep disturbances, worsening of mental health, and obesity. Compared with before the pandemic, the health status of boys and girls changed little. Eating habits tended to improve for both boys and girls.

## 1. Introduction

On 11 March 2020, the World Health Organization declared that the coronavirus disease 2019 (COVID-19) outbreak was a global pandemic [1]. Globally, 7561 million confirmed cases of COVID-19, including 68 million deaths, have been reported to the World Health Organization [2]. The COVID-19 pandemic greatly changed people’s daily lives, forcing countries to implement preventive measures such as lockdowns, isolation, and social distancing [3].

In Japan, infection with COVID-19 in children and adolescents is generally less severe and causes fewer deaths than in adults [4,5]. Less attention is paid to pediatric patients due to a milder course of the disease in children.

Problematic Internet use by high school students is an important public health issue [6]. Gaming disorders have been officially adopted in the 11th edition of the International Classification of Diseases [7]. Furthermore, screen time is traditionally associated with sedentary behavior and snacking, which may promote obesity and cardiovascular disease risk factors [8]. It also affects ocular health [9]. Therefore, appropriate Internet use, which is expected to develop increasingly in today’s society, is required. However, screen time is reportedly getting longer due to the COVID-19 pandemic [10,11]. In particular, high school students are in a critical period of self-management of the basic lifestyle habits they have acquired so far, and they need to be able to interact effectively with media, such as smartphones, games, and social networking services, while maintaining their mental and physical health. In Japan, extended screen time has been reported to have a negative impact on the physical and mental health of elementary school students [12].

However, no studies have been reported on the changes in leisure screen time before and during COVID-19 among Japanese private high school students who were not on lockdown and did not have remote classes, but for whom new lifestyles, such as social distancing, were introduced. Japanese high school students are between the ages of 15 and 18. Additionally, eating habits have been reported to worsen with more screen time, but there are no reports regarding this on Japanese high school students. The Health Behavior in School Children (HBSC) data for high school students before the pandemic were available, and we re-surveyed them during the COVID-19 pandemic to make a comparison.

This study aimed to determine whether adolescents’ leisure screen time differed during the COVID-19 pandemic compared to before the pandemic, and to identify factors that affect leisure screen time among Japanese high school students. Additionally, the HBSC questionnaire was used to investigate differences in eating habits and physical and mental health.

## 2. Methods

### 2.1. Participants

This was a longitudinal study conducted among adolescents aged 15–17 years attending a private high school in Niigata Prefecture, Japan. Participants answered the question twice; once in first grade and once in second grade. Adolescents were invited to participate in February 2020 and July 2021 (2020, *n* = 433, boys 41.1%; 2021, *n* = 417, boys 50.0%). Although the survey was administered to the same population, individual names and student ID numbers were not obtained, and paired tests were not conducted. Permission was obtained from the principal of the school and parental consent was obtained both times. 

### 2.2. Questionnaire

The questionnaire was adapted in part from the HBSC [13]. HBSC is a World Health Organization collaborative cross-national study of adolescent health and well-being. Founded in 1982, the survey is undertaken every four years using a self-report questionnaire. There are 51 countries and regions participating in this survey. Leisure screen time was assessed by asking participants how many hours, on average, they spent using an electronic device (e.g., LINE, Twitter, YouTube, and Internet) on weekdays and weekends. Specifically, 1: 0 h, 2: 0.5 h, 3: 1 h, 4: 2 h, 5: 3 h, 6: 4 h, 7: 5 h, 8: 6 h, 9: 7 h or more. Regarding eating habits, they were asked, on a scale of 1 to 8, how many times a week they ate certain food products (for example fruits, vegetables, sweets, soft drinks), with 1 denoting “I do not eat once a week” and 8 denoting “I eat at least twice a day.” The number of days of breakfast intake was answered separately for weekdays and weekends. Breakfast includes only one glass of milk or fruit juice. Next, happiness in life was assessed in a scale ranging from 0: worst possible life, to 9: best possible life. Participants were asked “How many days did you spent on social networks with friends” on a 5 point scale (1: never, to 5: daily). “Do your classmates enjoy being with you?” and “My classmates are kind and help me when I’m in trouble” were also asked using the 5-point scale (1: I strongly agree, 5: strongly disagree.) Participants reported the number of days they felt sad and lonely per week. Participants were asked to report the number of days per week they exercised for at least 60 min per day. Additionally, they were asked about physical and mental disorders (headache, stomach pain, feeling depressed, difficulty sleeping, irritability, and frequency of taking headache medicine, stomach medicine, and sleep medicine) looking back over the past month. This is because we hypothesized that longer screen time would have negative physical and mental effects and lead to bad eating habits, with reference to Szwarcwald et al. [14].

### 2.3. Statistical Analysis 

Statistical analysis was performed using the R statistical software (version 4.0.2.) [15]. A total of 813 subjects (boys, 45.3%) were included in the analysis. In calculating leisure screen time, responses of 7 h or more were calculated as 7 h. A *t*-test was used to compare screen time before and during the pandemic by sex. Binomial logistic regression models were used to examine the association between high (3 h) leisure screen time and health status and factors that predict leisure screen time. Models were run separately by sex. The dependent variable was “leisure screen time”. Since the average leisure screen time before the pandemic was 2.6 ± 1.4 h (mean ± SD), a screen time of less than 3 h was defined as 0 and a screen time of more than 3 h as 1. The independent variables were “more than 60 min of exercise (0 day to 7 days)”, “frequency of sweet intake”, “frequency of breakfast intake”, “friends help me”, “feeling down”, “lonely”, “best life”, “feeling sad”, “social networking with friends”, and “having fun with friends”. The explanatory variables were selected using the forward-backward stepwise method (Pin = Pout = 0.20). Akaike’s information criterion was used to select the model with the smallest value. The odds ratio (OR) with a 95% confidence interval (95% CI) was calculated for factors that increased leisure screen time. Multicollinearity was assessed using the variance inflation factor (VIF). A VIF exceeding 10 indicated serious multicollinearity, and values greater than 4.0 might be a cause for concern. The level of significance was set at 0.05 for all analyses.

### 2.4. Ethical Considerations

This study was conducted in accordance with the Declaration of Helsinki and approved by the Institutional Review Boards of Niigata University of Health and Welfare (approval number 18594-210412). The briefing document of the study was distributed, and the students took it home and were only asked for a response with the consent of their parents. The survey instructions included the purpose of the survey and the content and processing of the information obtained. All students were informed that participation was voluntary and that no disadvantage would be caused if they refused to participate.

## 3. Results

**Figure 1:**
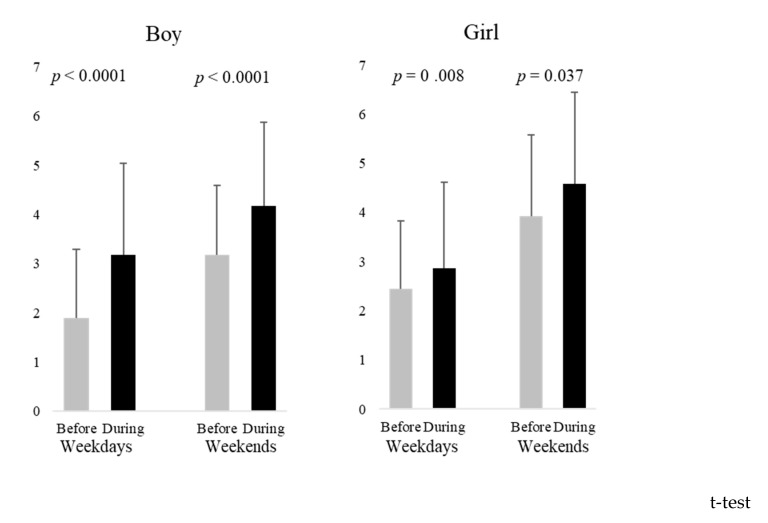
Comparison of the leisure screen time before and during the pandemic by sex.

The results of the leisure screen time comparison are shown in Figure 1. Both boys and girls experienced significantly more leisure screen time during the pandemic than before the pandemic, according to the results of the *t*-test. Leisure screen time on weekends was particularly long, with 4.2 h (SD = 1.7) for boys and 4.6 h (SD = 1.9) for girls. 

In the 2020 boys’ survey, “keeping in touch with friends on social networking sites”, “frequency of sweet intake”, “lonely”, and “having fun with friends” were found to be factors that were predictors of high (3 h) leisure screen time (Table 1). Also, in the 2021 survey, “keeping in touch with friends on social networking sites” and “best life” were predictors of high (3 h) leisure screen time (Table 2).

In the girls’ survey, the number of days those girls “exercise for 60 min or more” was one of the factors that reduced leisure screen time in 2020 and 2021 (Table 3 and Table 4). Additionally, “keeping in touch with friends on social networking sites” increased leisure screen time only in the 2021 survey. The largest VIF were 2.67 for boys and 2.27 for girls in 2020, and 2.40 for boys and 2.44 for girls in 2021.

Differences in eating habits before and during the pandemic are shown in Table 5. Two items changed significantly in boys. The first was an increase in the frequency of fruit consumption (*p* = 0.013), and the second was a decrease in the frequency of sweets consumption (*p* < 0.001). However, the frequency of consumption of vegetables (*p* = 0.029), sweets (*p* = 0.013), soft drinks (*p* < 0.001), and weekend breakfast decreased significantly for girls. Table 6 shows the mental and physical health conditions and frequency of taking medication. For both boys and girls, no changes in physical condition or frequency of taking medications were observed before and during the pandemic. Girls tended to have less headache frequency during the pandemic (*p* = 0.059). 

## 4. Discussion

This study aimed to determine whether adolescents’ leisure screen time differed during the COVID-19 pandemic compared to before the pandemic, and to identify the factors that affect leisure screen time among Japanese high school students. Additionally, this study aimed to investigate changes in mental and physical conditions and dietary habits. The leisure screen time of Japanese high school students, both boys and girls, differed between during the COVID-19 pandemic compared to beforehand. Different factors differed the leisure screen time for boys and girls. Additionally, no significant deterioration was observed in physical and mental health. The impact of the pandemic on adolescent eating habits differed in boys and girls.

Similar to the findings reported by Bahkir et al., Japanese high school students have longer leisure screen time since the start of the pandemic [9]. Hong Kong children’s leisure screen time increased from 4.4 h before the pandemic to 5.6 h during the pandemic [16]. A recent report in the United States showed that children’s non-academic screen time increased from 3.8 h per day to 7.7 h per day during the COVID-19 pandemic [17]. In Brazil, 46.4% of adolescents reported more than 6 h of leisure screen time [14].

Excessive adolescents’ screen use has been associated with physical and mental health risks [4,18,19,20]. Both boys and girls had more leisure screen time during the pandemic in 2021 than in 2020. In particular, on holidays, the average leisure screen time was 4.2 h for boys and 4.6 h for girls. The leisure screen time was longer on weekends than on weekdays. This finding is consistent with that reported by Peddie et al. in New Zealand [21]. Long screen time has been reported to be associated with sleep disturbance [22], loneliness, and mental health [23,24] and is presumed to be one of the points to watch out for during the pandemic. However, about 50% of students in Japan had less than 2 h of leisure screen time. The reason why the leisure screen time was shorter in Japan than that in other countries is that, although many extracurricular activities (both physical and cultural) were canceled, is that the students could attend high school and no remote classes were offered.

For boys, being on social networking sites with friends resulted in longer leisure screen time in both 2020 and 2021 (Table 2 and Table 3), but other factors that increased leisure screen time differed between the two years. Before the pandemic, “not having a good time with friends” influenced the extension of leisure screen time, but during the pandemic, “feeling that life is not the best right now” was a factor that influenced the extension of leisure screen time. Since screen time was longer when feeling depressed during the pandemic, it is inferred that, for boys, higher leisure screen time may be an indicator of feeling unwell.

For girls, not exercising for more than 60 min was a factor that affected leisure screen time (Table 3 and Table 4). Since regular exercise has been reported to decrease depressive and anxiety symptom levels and improve sleep quality [25,26], the excessive higher leisure screen time during the pandemic can be curtailed by allowing time for exercise, and will, therefore, further improve physical and mental health.

The pre-pandemic results are consistent with those reported by George et al., who stated that smartphone addiction was associated with loneliness in adolescence, but the results during the pandemic were different [27]. It was inferred that people were in contact with the media during the pandemic due to different factors from those during normal times [28]. Loneliness was not a factor that differed leisure screen time among girls. These results indicate that the factors affecting leisure screen time behavior are different between boys and girls.

The pandemic reduced the frequency of vegetable consumption among girls (Table 5). Additionally, the frequency of breakfast intake on holidays and drinking soft drinks decreased. This indicated that the pandemic affected eating habits even in adolescents in Japan who did not have lockdown or remote classes. These findings are consistent with the study by López-Bueno et al. on Spanish children and reports from Poland [29,30]. Reports from China and the Arab world have shown that regular fruit and vegetable consumption has a positive impact on emotional well-being [20,31]. Therefore, increasing the frequency of fruit and vegetable consumption might lead to a sense of well-being among the adolescent students in this study. Although it has been reported that sweet intake increased [32], in this study, the frequency of sweet intake decreased significantly. The reason for this may be that lockdowns and remote classes did not increase as in other countries. On the other hand, in boys, the frequency of fruit intake increased significantly (*p* = 0.013) and the frequency of sweets intake decreased significantly (*p* = 0.033, Table 5). We speculated that the pandemic had a different impact on adolescent eating habits for males and females.

We hypothesized that longer leisure screen time would be detrimental to physical and mental health. However, this hypothesis was not supported by this study. No significant physical or mental deterioration due to the COVID-19 pandemic was observed in Japanese adolescents (Table 6). In a study of Japanese elementary school children, Ueno et al. reported that long screen time negatively affected both the physical and mental health of the children [12]. In addition, there are reports of a relationship with depression [22,23] and with sleep disorders [16], but the frequency of headache, stomach pain, irritability, and sleep disorders did not change in this study. We speculate that this is because the length of leisure screen time during the pandemic was not as long in Japan as that reported in other countries. The lack of lockdowns and the fact that pre-pandemic life was maintained to a certain extent may have contributed to this outcome.

This study has several limitations. Because this study was conducted at a private high school in a rural city far from the metropolitan area, similar results may not be obtained for adolescents in the metropolitan area or for high school students in public high schools. A few students did not respond to the first and second surveys, but because we did not provide their names and student ID numbers, it was not possible to match all of them perfectly. This study consisted of questions taken from the HBSC questionnaire; the HBSC screen time question had a maximum value of 7 h selected, and it was not possible to ascertain the amount of time beyond that. Therefore, screen time of more than 7 h was calculated as 7 h, so it cannot be denied that the actual average screen time may be longer.

The COVID-19 pandemic in Japan has subsided since the survey was conducted in July 2021, and social life is returning to pre-pandemic levels. In high school life, extracurricular activities other than classes (e.g., sports festival, cultural festivals, excursions, and club activities) are becoming possible. Therefore, in the future, we would like to investigate whether leisure screen time returns to pre-pandemic levels when the pandemic subsides, and if leisure screen time is still long, we would like to devise an appropriate teaching method so as not to cause health damage and deterioration of eating habits.

This longitudinal study of adolescents attending a private high school in a rural Japanese city is one of the few studies to compare the same population before and during the COVID-19 pandemic. This study showed that leisure screen time was longer during the pandemic than before the pandemic, but the leisure screen time was shorter in Japan than in other countries. We speculated that the reason for this was that they could attend high school and that it was important for them to maintain their pre-pandemic lifestyle as much as possible to maintain their health.

## 5. Conclusions

Leisure screen time has increased for Japanese high school students due to the COVID-19 pandemic. In addition to being connected to friends on social networks, boys reported “not feeling great about life” as a factor that differed leisure screen time during the pandemic from pre-pandemic, suggesting that negative emotions influenced the higher leisure screen time. For girls, the pandemic also had a significant impact on their leisure screen time, and “exercising less than 60 min a day” increased leisure screen time regardless of the pandemic. It is speculated that factors other than those surveyed may have contributed to extended leisure screen time due to the pandemic. Longer screen time should be carefully monitored because it can lead to sleep disturbances, worsening of mental health, and obesity. Eating habits tended to improve for boys. Compared with the pre-pandemic period, the health status of both boys and girls were not changed. 

## Figures and Tables

**Table 1:** Predictors of high (3 h) leisure screen time (the 2020 boys’ survey).

	Partial Regression Coefficient	SD	OR	95% CI	*p*-Value
Intercept	0.718	0.345	2.050	[0.037, 1.399]	0.039
Frequency of sweet intake	0.052	0.024	1.054	[0.004, 0.101]	0.034
Lonely	−0.096	0.045	0.909	[−0.186, −0.006]	0.037
Feeling sad	0.068	0.045	1.070	[−0.022, 0.157]	0.137
Social networking with friends	0.070	0.025	1.072	[0.020, 0.120]	0.007
Having fun with friends	−0.130	0.035	0.878	[−0.198, −0.061]	<0.001
Frequency of breakfast intake (weekdays)	−0.028	0.020	0.972	[−0.067, 0.010]	0.151
Friends help me	−0.078	0.041	0.925	[−0.158, 0.002]	0.055

SD: standard deviation; OR: odds ratio; 95% CI: 95% confidence interval. *n* = 172.

**Table 2:** Predictors of high (3 h) leisure screen time (the 2021 boys’ survey).

	Partial Regression Coefficient	SD	OR	95% CI	*p*-Value
Intercept	0.132	0.311	1.141	[−0.486, 0.750]	0.674
Frequency of sweet intake	0.036	0.027	1.037	[−0.017, 0.089]	0.178
Feeling down	0.074	0.038	1.077	[−0.001, 0.149]	0.054
Best life	−0.043	0.020	0.958	[−0.082, −0.004]	0.032
Social networking with friends	0.077	0.024	1.080	[0.029, 0.125]	0.002

SD: standard deviation; OR: odds ratio; 95% CI: 95% confidence interval. *n* = 19.6

**Table 3:** Predictors of high (3 h) leisure screen time (the 2020 girls’ survey).

	Partial Regression Coefficient	SD	OR	95% CI	*p*-Value
Intercept	0.635	0.425	1.887	[−0.204, 1.474]	0.137
More than 60 min of exercise	−0.038	0.015	0.963	[−0.069, −0.006]	0.018
Frequency of sweet intake	0.048	0.028	1.050	[−0.007, 0.102]	0.085
Lonely	0.053	0.040	1.054	[−0.027, 0.132]	0.194
Best life	−0.028	0.018	0.973	[−0.063, 0.007]	0.118
Frequency of breakfast intake (weekdays)	0.026	0.049	1.026	[−0.071, 0.123]	0.074

SD: standard deviation; OR: odds ratio; 95% CI: 95% confidence interval. *n* = 24.7

**Table 4:** Predictors of high (3 h) leisure screen time (the 2021 girls’ survey).

	Partial Regression Coefficient	SD	OR	95% CI	*p*-Value
Intercept	0.325	0.413	1.383	[−0.490, 1.139]	0.325
More than 60 min of exercise	−0.042	0.016	0.959	[−0.073, −0.011]	0.008
Lonely	0.059	0.046	1.061	[−0.033, 0.151]	0.200
Social networking with friends	0.056	0.027	1.067	[0.002, 0.110]	0.042

SD: standard deviation; OR: odds ratio; 95% CI: 95% confidence interval. *n* = 19.8

**Table 5:** Difference between pre-pandemic and pandemic high school students’ eating habits.

	Boy			Girl		
	2020	2021	*p*-Value	2020	2021	*p*-Value
	*n* = 172	*n* = 196		*n* = 247	*n* = 198	
Fruits	3.4 ± 1.5	3.8 ± 1.5	0.013	3.6 ± 1.7	3.7 ± 1.5	0.860
Vegetables	5.3 ± 1.5	5.4 ± 1.3	0.449	5.8 ± 1.2	5.5 ± 1.3	0.029
Sweet	4.0 ± 1.6	3.7 ± 1.5	0.033	4.6 ± 1.4	4.1 ± 1.5	<0.001
Soft drink	3.9 ± 1.6	3.8 ± 1.6	0.629	3.2 ± 1.8	2.8 ± 1.5	0.029
Weekday breakfast	5.5 ± 2.3	5.5 ± 1.2	0.062	5.6 ± 1.2	5.6 ± 1.0	0.924
Weekend breakfast	3.6 ± 1.8	3.0 ± 1.3	0.099	3.5 ± 1.8	2.7 ± 1.3	<0.001

Mean ± SD.

**Table 6:** Physical and mental health conditions and frequency of taking medication.

	Boy			Girl		
	2020	2021	*p*-Value	2020	2021	*p*-Value
	*n* = 172	*n* = 196		*n* = 247	*n* = 198	
Headache	4.1 ± 1.3	4.1 ± 1.1	0.818	3.9 ± 1.4	3.7 ± 1.3	0.059
Stomach pain	4.4 ± 1.2	4.5 ± 2.2	0.416	4.4 ± 1.1	4.2 ± 1.1	0.148
Feeling depressed	3.6 ± 1.4	3.7 ± 1.3	0.558	3.4 ± 1.4	3.3 ± 1.3	0.424
Irritability	3.8 ± 1.4	3.6 ± 1.3	0.384	3.4 ± 1.4	3.3 ± 1.2	0.460
Difficulty sleeping	4.5 ± 1.2	4.4 ± 1.1	0.654	4.5 ± 1.0	4.5 ± 1.0	0.656
Headache medications taken	1.2 ± 0.6	1.3 ± 0.8	0.140	1.6 ± 0.9	1.6 ± 0.9	0.800
Gastric medications taken	1.2 ± 0.6	1.2 ± 0.6	0.866	1.3 ± 0.7	1.2 ± 0.6	0.298
Insomnia medication taken	1.0 ± 0.3	1.1 ± 0.6	0.151	1.1 ± 0.5	1.1 ± 0.4	0.582

Mean ± SD.

## Data Availability

The data that support the findings of this study are available from the corresponding author.
